# The first succinylome profile of *Trichophyton rubrum* reveals lysine succinylation on proteins involved in various key cellular processes

**DOI:** 10.1186/s12864-017-3977-y

**Published:** 2017-08-04

**Authors:** Xingye Xu, Tao Liu, Jian Yang, Lihong Chen, Bo Liu, Candong Wei, Lingling Wang, Qi Jin

**Affiliations:** MOH Key Laboratory of Systems Biology of Pathogens, Institute of Pathogen Biology, Chinese Academy of Medical Sciences & Peking Union Medical College, No. 6, Rongjing East Street, BDA, Beijing, 100176 China

**Keywords:** Post-translational modification (PTM), Lysine succinylation (Ksucc), Dermatophyte, *Trichophyton rubrum* (*T. rubrum*)

## Abstract

**Background:**

Dermatophytes, the most common cause of fungal infections, affect millions of individuals worldwide. They pose a major threat to public health because of the severity and longevity of infections caused by dermatophytes and their refractivity to therapy. *Trichophyton rubrum* (*T. rubrum*), the most common dermatophyte species, is a promising model organism for dermatophyte research. Post-translational modifications (PTMs) have been shown to be essential for many biological processes, particularly in the regulation of key cellular processes that contribute to pathogenicity. Although PTMs have important roles, little is known about their roles in *T. rubrum* and other dermatophytes. Succinylation is a new PTM that has recently been identified. In this study, we assessed the proteome-wide succinylation profile of *T. rubrum*. This study sought to systematically identify the succinylated sites and proteins in *T. rubrum* and to reveal the roles of succinylated proteins in various cellular processes as well as the differences in the succinylation profiles in different growth stages of the *T. rubrum* life cycle.

**Results:**

A total of 569 succinylated lysine sites were identified in 284 proteins. These succinylated proteins are involved in various cellular processes, such as metabolism, translation and epigenetic regulation. Additionally, 24 proteins related to pathogenicity were found to be succinylated. Comparison of the succinylome at the conidia and mycelia stages revealed that most of the succinylated proteins and sites were growth-stage specific. In addition, the succinylation modifications on histone and ribosomal proteins were significantly different between these two growth stages. Moreover, the sequence features surrounding the succinylated sites were different in the two stages, thus indicating the specific recognition of succinyltransferases in each growth phase.

**Conclusions:**

In this study, we explored the first *T. rubrum* succinylome, which is also the first PTM analysis of dermatophytes reported to date. These results revealed the major roles of the succinylated proteins involved in *T. rubrum* and the differences in the succinylomes between the two major growth stages. These findings should improve understanding of the physiological and pathogenic properties of dermatophytes and facilitate future development of novel drugs and therapeutics for treating superficial fungal infections.

**Electronic supplementary material:**

The online version of this article (doi:10.1186/s12864-017-3977-y) contains supplementary material, which is available to authorized users.

## Background

Post-translational modifications (PTMs) are an efficient strategy to extend the diversity of protein functions, and they play important roles in regulating multiple cellular events in both prokaryotic and eukaryotic organisms [1, 2]. Lysine is a frequently modified amino acid residue because it is important in protein folding. The modification of lysine promotes the transformation of the spatial structures and chemical properties of proteins, hence affecting protein functions [3, 4]. Lysine succinylation (Ksucc) was first identified in *E. coli* in 2011 [4]. Ksucc-modified proteins have been reported to participate in a wide range of important cellular processes, such as the regulation of metabolism [5].

Dermatophytes are a group of filamentous fungi that cause infections and are among the most common causes of human diseases, affecting nearly 20% of the global population [6]. Examples of dermatophyte-caused infections are tinea pedis and tinea capitis. Furthermore, deep dermatophytosis caused by dermatophyte infections has also been reported, in which the infection penetrates the skin barrier and reaches internal tissues and organs [7]. Although dermatophyte-caused infections rarely cause death, their prevalence, high incidence, difficulty to treat and contribution to morbidity represent a significant unsolved global public health problem [8].

*T. rubrum* is the major causative agent of dermatomycoses, which accounts for more than 60% of dermatophyte infections [9]. *T. rubrum* causes widespread infections because of its good vitality, that it remains viable for more than 6 months in the environment [10]. *T. rubrum* has a non-sexual life-cycle, comprising a tear-shaped conidia stage and a hyaline septate mycelia stage. Conidia protect the *T. rubrum* genome during adverse environmental conditions, and infection is triggered when the conidia adhere to the corneum of the skin and form mycelia. The longitudinally growing mycelia penetrate deep into the corneum and cause skin damage [11, 12]. Because of its growth features and prevalence, *T. rubrum* has been considered a good model system to use in the study of human pathogenic filamentous fungi [11, 13].

Although a PTM analysis has not been conducted for *T. rubrum* and other dermatophytes, accumulating evidence has suggested that PTMs are essential for the growth and development of fungi, particularly in the regulation of key cellular processes that contribute to fungal pathogenicity. For example, the role of *N*-glycosylation in fungal pathogenesis has been validated. The α-1, 6-mannosyltransferase Och1 initiates the formation of a distinct branch on the *N*-glycan core, which allows for the subsequent addition of mannosylated outer chains. Och1 mutants in *C. albicans* display major cell wall defects and decreased virulence [14]. In another case, protein *O*-mannosyltransferases (PMTs) initiate *O*-mannosyl glycan biosynthesis in the endoplasmic reticulum (ER). Triple PMT mutants are lethal in *S. cerevisiae* [15], and the loss of PMTs leads to attenuated virulence in both *C. albicans* and *C. neoformans* [16–19]. In addition, the regulation of ubiquitination in fungal pathogens is involved in stress adaptation, metabolism, morphogenesis and other developmental processes. The pathways regulated by ubiquitination are fundamentally essential processes that are important for fungal virulence [20]. Therefore, PTM analysis in *T. rubrum* should improve understanding of the regulatory strategies used in essential physical processes and their contributions to infections. This information should be informative for the development of drugs to treat infections caused by these clinically important fungi.

This study sought to systematically identify the succinylated sites and proteins in *T. rubrum* and to reveal the roles of the succinylated proteins involved in cellular processes and the differences in the succinylation profiles in different growth stages of the *T. rubrum* life cycle. A total of 569 succinylated sites in 284 proteins were identified. These succinylated proteins are primarily involved in metabolism and translation, especially in pathogenicity-related processes. In addition, most of the succinylated proteins and sites have been shown to be growth-stage specific. Our study provides the first reported *T. rubrum* succinylome and is also the first PTM analysis of dermatophytes reported to date. Thus, this work represents great progress in research on these medically important fungi.

## Results and discussion

### Proteome-wide identification of lysine succinylation in *T. rubrum*

**Fig. 1:**
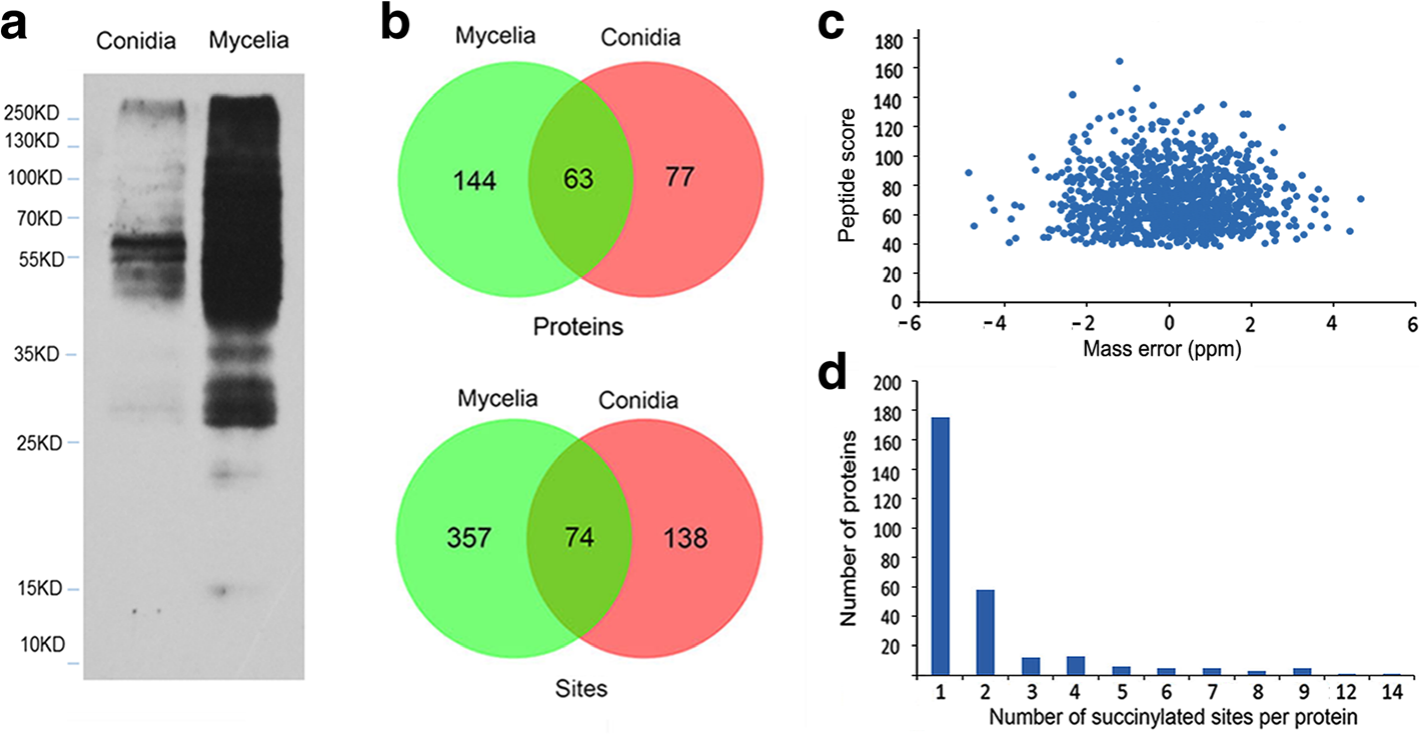
The identification of lysine succinylation in *T. rubrum.* **a** Western blot analysis. **b** A Venn diagram of the succinylated sites and proteins. **c** The distribution of mass error. **d** The number of succinylated sites per protein plotted against the number of proteins

Western blotting analysis showed that a large number of proteins were modified by succinylation and the succinylated proteins were more abundant in the mycelia stage than in the conidia stage (Fig. 1a). In the subsequent LC-MS/MS analysis, 569 succinylated sites in 284 proteins were identified in the two stages of the *T. rubrum* life cycle. Two hundred and twelve succinylated sites in 140 proteins were identified in the conidia stage, and 431 succinylated sites in 207 proteins were identified in the mycelia stage (Fig. 1b). The scores of all the identified peptides were above 40. The mass error for most peptides was <4 ppm (Fig. 1c), thus indicating that the mass accuracy of the MS data was sufficient for further analyses [21]. The succinylated proteins and sites identified in each replicate for the conidia and mycelia stages are shown in Additional file 1: Figure S1 and Additional file 2: Table S1. In the three biological repeats, many more succinylated proteins and sites were identified, notably some low-abundance succinylated proteins and sites. In order to extensively investigate the function of the succinylated proteins identified in *T. rubrum*, the succinylated proteins and sites identified in any replicate were subjected to further study. Most of the identified proteins (175 proteins) possessed only one succinylated site, whereas 58 proteins possessed two sites, and 51 proteins possessed three or more succinylated sites (Fig. 1d).

### Functional annotation and subcellular localization of the succinylated proteins

**Fig. 2:**
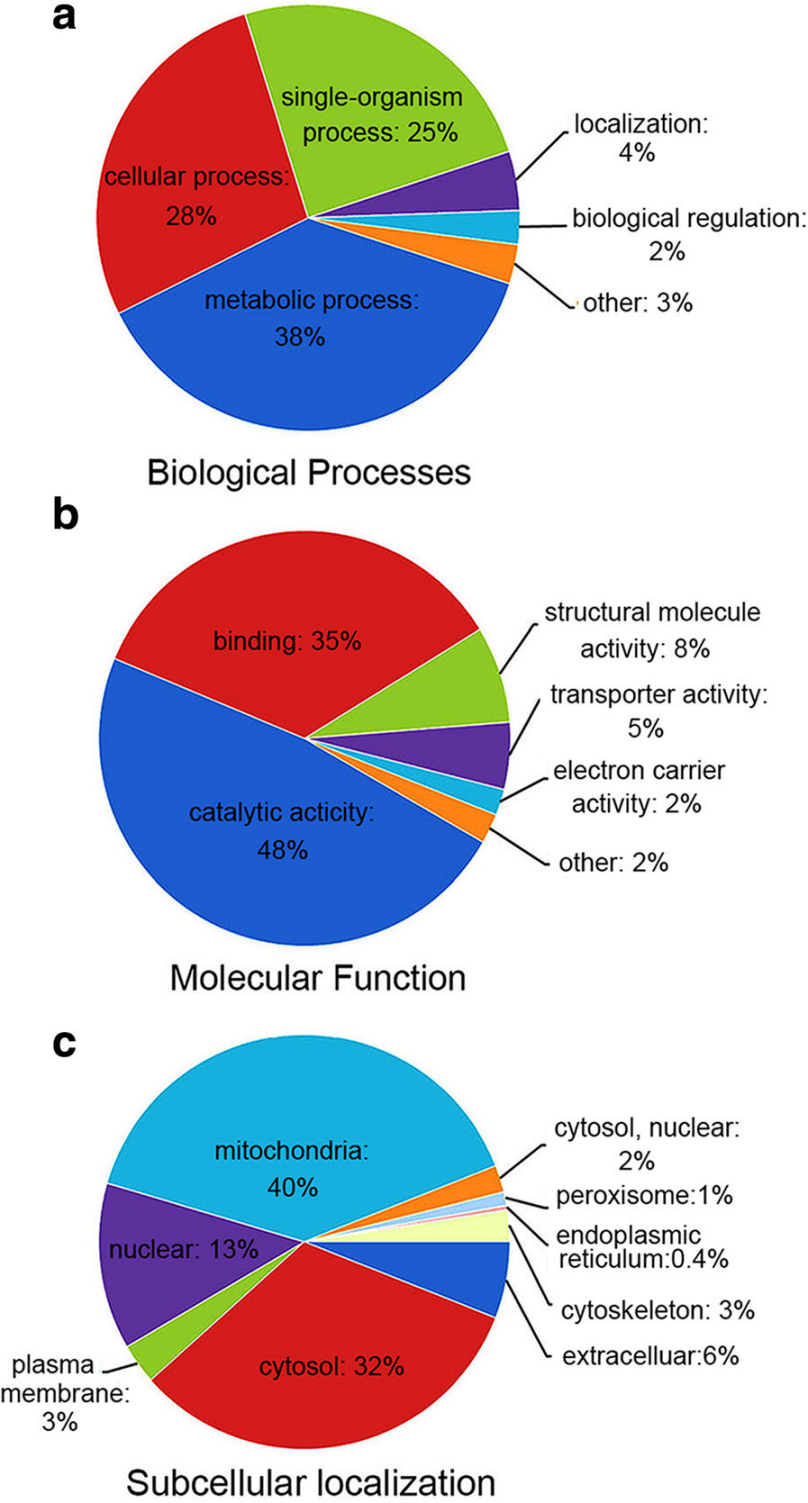
The classification of the succinylated proteins. **a** The GO characterization of the identified succinylated proteins on the basis of biological processes. **b** The GO characterization of the identified succinylated proteins on the basis of molecular function. **c** The distribution of the subcellular localization of the succinylated proteins predicted with WoLF PSORT

We performed a functional classification of all identified succinylated proteins by using GO analysis (Additional file 3: Table S2). As shown in the classification of the biological processes represented by the succinylated proteins (Fig. 2a), the three largest protein groups were involved in metabolism (38%), followed by cellular processes (28%) and single-organism processes (25%). As shown in the molecular function classification (Fig. 2b), the two largest protein groups were catalytic activity (48%) and binding (35%), which were consistent with the classification of the biological processes mentioned above.

According to the results of the subcellular localization analysis (Fig. 2c), most succinylated proteins were localized to the mitochondria (40%) (Additional file 4: Table S3). This percentage was higher than the percentage in yeast (8%). However, in HeLa cells and mouse liver, these ratios were much higher, 45 and 70%, respectively [22]. Succinylation tends to occur in the mitochondria because the mitochondria are the major provenance for succinyl-CoA and succinate from the tricarboxylic acid (TCA) cycle or odd-numbered fatty acid oxidation [5]. In addition, in mouse liver, mitochondrial proteins are more frequently succinylated at multiple sites than non-mitochondria proteins [22]. Our data showed the same bias, in that 47% of mitochondrial proteins contained multiple succinylated sites (≥ 2 succinylated sites) compared with 33% of non-mitochondrial proteins (Additional file 5: Table S4). However, the small succinyl metabolites (like succinyl-CoA and succinate) can traverse the mitochondrial membrane or be formed outside of mitochondria [22]. For example, succinate could be formed in the cytoplasm as a side product by ketoglutarate-dependent enzymes [23]. It has been suggested that in *S. cerevisiae*, *H. sapiens* and *M. musculus* succinyl metabolites drive succinylation in the cytoplasm and nucleus [22]. In *T. rubrum*, 32% of the succinylated proteins are located in the cytosol, and 13% of succinylated proteins were found to be located in the nucleus.

### Enrichment analysis of the succinylated proteins

**Fig. 3:**
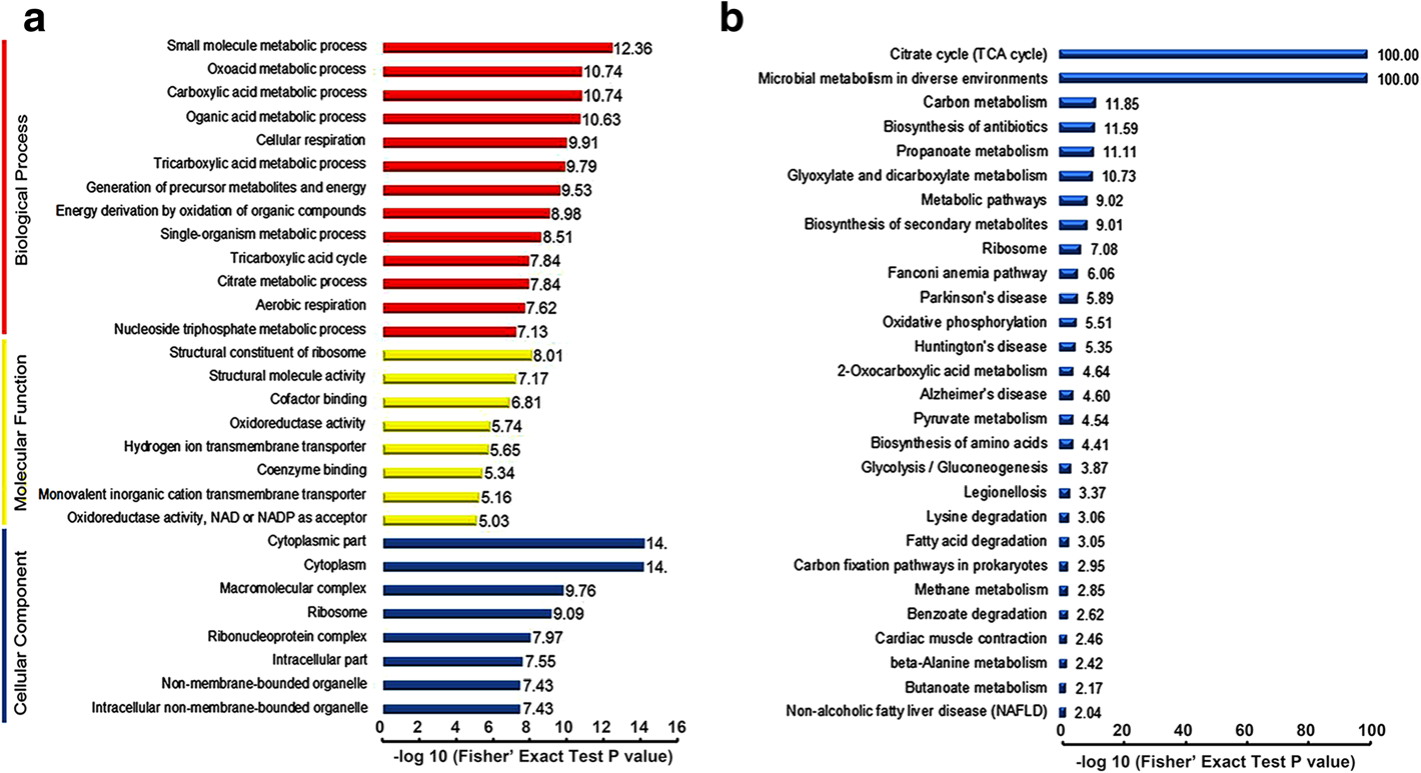
The enrichment analysis of the succinylated proteins. **a** The GO enrichment based on the biological processes (*p* < 10^−7^), molecular functions (*p* < 10^−5^) and cellular components (*p* < 10^−7^) of the proteins. **b** KEGG pathway enrichment (*p* < 0.01)

We performed GO and KEGG enrichment analyses to further elucidate the types of proteins that are targets for succinylation. On the basis of GO enrichment for biological processes, the succinylated proteins were primarily involved in small molecule metabolic processes, oxoacid metabolic processes, carboxylic acid metabolic processes and organic acid metabolic processes, as shown in Fig. 3a. For the molecular function enrichment analysis, the structural constituents of the ribosome, structural molecule activity, cofactor binding and oxidoreductase activity were significantly enriched. Thus, a large percentage of the succinylated proteins participate in metabolic and translational relating roles. The enrichment analysis of the cellular components showed that the ribosome and ribonucleoprotein complex were significantly enriched, thus suggesting that succinylated proteins are closely related to translation and supporting the above conclusion. (Additional file 6: Table S5).

On the basis of KEGG pathway enrichment analysis (Additional file 7: Table S6), 121 succinylated proteins were classified as being involved in metabolic pathways. As shown in Fig. 3b, proteins associated with processes such as the TCA cycle, carbon metabolism, oxidative phosphorylation, the ribosome, glycolysis/gluconeogenesis were significantly enriched.

**Fig. 4:**
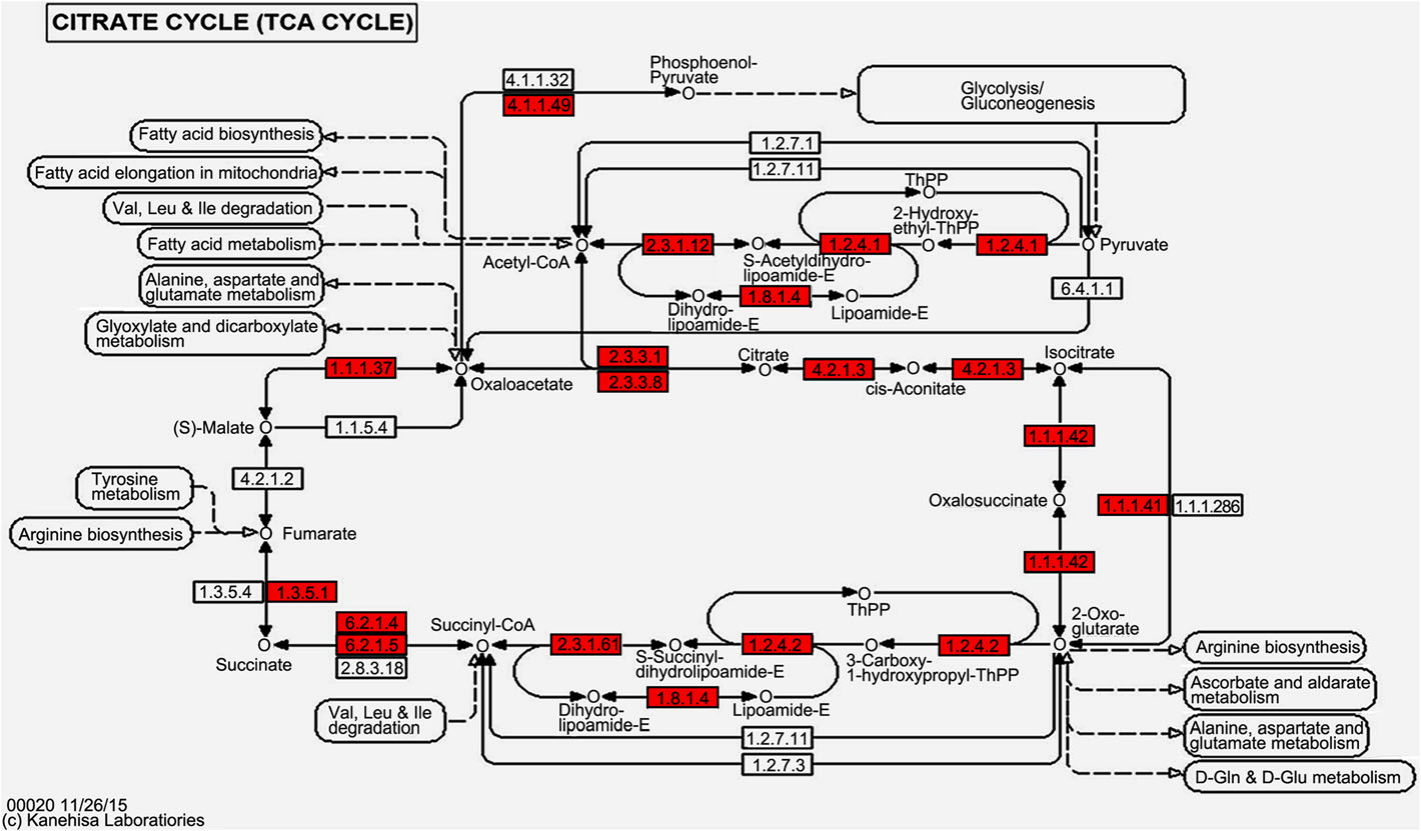
The succinylated proteins involved in the TCA cycle. The succinylated proteins are highlighted in *red*

The TCA cycle is present in a wide variety of aerobic organisms. In our study, nearly every enzyme in the TCA cycle was succinylated, and most of these enzymes were succinylated at multiple sites (Fig. 4). The succinylated enzymes and modified sites are listed in Additional file 8: Table S7. For example, the enzyme aconitate hydratase (ACO), which catalyzes the conversion of citrate to isocitrate, is succinylated at 9 different sites. Another enzyme, isocitrate dehydrogenase (IDH), catalyzes the conversion of isocitrate to 2-oxoglutarate, the rate-limiting step of the TCA cycle. In our study, we identified 7 succinylated sites in IDH. In turn, lysine succinylation is also affected by the enzymes involved in the TCA cycle. For example, succinyl-CoA ligase utilizes succinate and CoA to form succinyl-CoA, which non-enzymatically catalyzes protein succinylation. In yeast, loss of α-ketoglutarate dehydrogenase (Kgd1) and succinyl-CoA ligase (LSC1) has been suggested to affect global succinylation levels [22]. In *T. rubrum*, two α-ketoglutarate dehydrogenases (SucA and SucB) and two succinyl-CoA ligases (LSC1 and SucD) were succinylated, thus suggesting a potential effect of succinylation on the functions of these enzymes.

Oxidative phosphorylation (OPP) is another pathway that occurs in the mitochondria of most eukaryotes. OPP is a highly efficient method for releasing the energy that is used to reform ATP. During OPP, electrons are transferred from electron donors to electron acceptors in redox reactions. In eukaryotes, five main protein complexes are involved in these redox reactions. In our study, succinylation was observed on several subunits of every protein complex involved in OPP (Additional file 1: Figure S2). For example, eight subunits of F-type ATPase (complex V) were succinylated, most of which were succinylated at multiple sites. The F-type ATPase alpha subunit (ATP1) contained 14 succinylated sites, the most of any protein. Three succinylated sites in ATP1 (K164, K233, and K430) were conserved in *M. musculus* (mouse) [24]. In previous studies, ATP1 have been found to be heavily modified at lysine residues, and the most common types of modifications were acetylation and ubiquitination [25]. These results suggest that succinylation and other types of modifications may work together to affect ATP1 function. The succinylated enzymes involved in OPP and the modified sites are listed in Additional file 9: Table S8.

### Succinylated proteins related to pathogenicity

We identified 24 succinylated proteins related to pathogenicity in *T. rubrum* or homologous proteins involved in virulence in other fungi (Table 1). Secreted proteases, which digest hard keratin tissues during infection, are important in the virulence of dermatophytes. Eight secreted proteases were identified to be succinylated, including aminopeptidase, aspartic endopeptidase Pep2, leucine aminopeptidase 1, leucine aminopeptidase 2, subtilisin-like protease, Peptidase S41 family protein, tripeptidyl-peptidase SED2 and carboxypeptidase S1. In addition to secreted proteases, the mdr2-encoded ABC multidrug transporter (succinylated at K361 and K368) and AcuE-encoded malate synthase (succinylated at K161, K319, K483, K486 and K501) are also involved in dermatophyte infection [26–28]. Moreover, two Rho-type GTPases identified in our study were succinylated, Rho GTPase Rho1 and Rho-GDP dissociation inhibitor (Rho-GDI). Rho-type GTPases regulate many fundamental growth processes, such as cytoskeletal arrangement, vesicle trafficking, cell wall biosynthesis and polarized growth, and they have also been implicated in fungal infection [29–31]. Furthermore, heat shock proteins (Hsps) have also been implicated in fungal pathogenicity. In addition to their roles as chaperone proteins, Hsps have specific roles in fungi, such as dimorphic transition, drug resistance and virulence. For example, Hsp90, which is involved in morphogenesis, antifungal resistance and fungal pathogenicity, is considered a potential target for antifungal therapy [32]. The four Hsps identified in our study, Hsp31, Hsp60, Hsp70 and Hsp90, were heavily succinylated. These key proteins that are critical for pathogenicity were identified as succinylation targets. Further experiments are needed to investigate whether succinylation plays a role in *T. rubrum* pathogenicity.

**Table 1:** The identified succinylated proteins related to pathogenicity in fungi

Protein names	Species^a^	Protein accessions	Succinylated lysine sites
14–3-3 family protein	*C. albicans*	TERG_01614T0	K51
		TERG_01614T1	K28
		TERG_06816T0	K49, K117, K122
ABC multidrug transporter Mdr2	*T. rubrum, C. albicans, A. nidulans*	TERG_06399T0	K361, K368
ABC transporter	*C. albicans, A. fumigatus, C. neoformans,* etc.	TERG_04224T0	K12
Aminopeptidase	*T. rubrum, A. benhamiae, M. canis, A. fumigatus*	TERG_06767T0	K645
		TERG_06767T2	K272, K518
		TERG_12154T0	K680, K946
Hsp60-like protein	*C. albicans, H. capsulatum*	TERG_04141T0	K48, K75, K89, K130, K277, K282, K430, K437
AhpC/TSA family thioredoxin peroxidase	*A. fumigatus, C. albicans, C. neoformans*	TERG_05504T0	K66, K142
Aspartic endopeptidase Pep2	*A. benhamiae, M. canis, C. albicans, A. fumigatus*	TERG_06704T2	K163
Calnexin	*A. fumigatus*	TERG_07527T0	K155, K203
Catalase	*A. nidulans, C. albicans, C. neoformans*	TERG_02005T0	K491
Glutathione S-transferase GstA	*A. fumigatus*	TERG_00370T0	K202
G-protein complex beta subunit CpcB	*C. heterostrophus, V. dahliae*	TERG_00783T0	K56
Heat shock protein 70 (Hsp70)	*C. albicans, H. capsulatum, C. neoformans*	TERG_03206T1	K37, K58, K137, K165, K222, K223, K276, K302, K331, K528, K545, K571
		TERG_06505T0	K91, K157, K244, K326, K422, K511
		TERG_06505T2	K422
		TERG_03037T0	K134, K102, K131, K323, K367
		TERG_01002T0	K482
		TERG_01883T0	K361
Leucine aminopeptidase 1	*T. rubrum, A. benhamiae, M. canis, A. oryzae, A. fumigatus*	TERG_05652T0	K116
Leucine aminopeptidase 2	*T. rubrum, A. benhamiae, M. canis, A. oryzae, A. fumigatus*	TERG_08405T1	K17, K46, K380, K416
Subtilisin-like protease	*A. fumigatus, T. rubrum, A. benhamiae, M. canis and other dermatophyte species*	TERG_12591T0	K255
Malate synthase AcuE	*C. albicans, A. benhamiae*	TERG_01281T0	K161, K319, K483, K486, K501
Molecular chaperone Mod-E/Hsp90	*F. graminearum, C. albicans, A. fumigatus*	TERG_06963T0	K171, K382, K385, K436, K479, K515, K550, K559, K565
Peptidase S41 family protein	*A. fumigatus, T. rubrum, A. benhamiae, M. canis*	TERG_08195T1	K490
Peptidyl-prolyl cis-trans isomerase	*C. albicans, C. neoformans*	TERG_01573T0	K36, K75, K120, K113
		TERG_06858T0	K67, K75, K88, K126
Probable chaperone protein Hsp31 homologue, putative	*C. albicans*	TERG_00228T0	K159
Rho GTPase Rho1	*C. albicans, C. neoformans*	TERG_07578T0	K155
Rho-gdp dissociation inhibitor	*C. neoformans*	TERG_05090T3	K125
		TERG_05090T4	K122
		TERG_05090T5	K125
Tripeptidyl peptidase SED2	*A. fumigatus, T. rubrum*	TERG_00619T0	K340
carboxypeptidase S1, putative	*T. rubrum, A. benhamiae, M. canis, A. fumigatus*	TERG_08255T1	K490

^a^The column “Species” indicates the organisms in which the proteins involved in pathogenicity have been reported

### Secondary structure properties of succinylated lysine

**Fig. 5:**
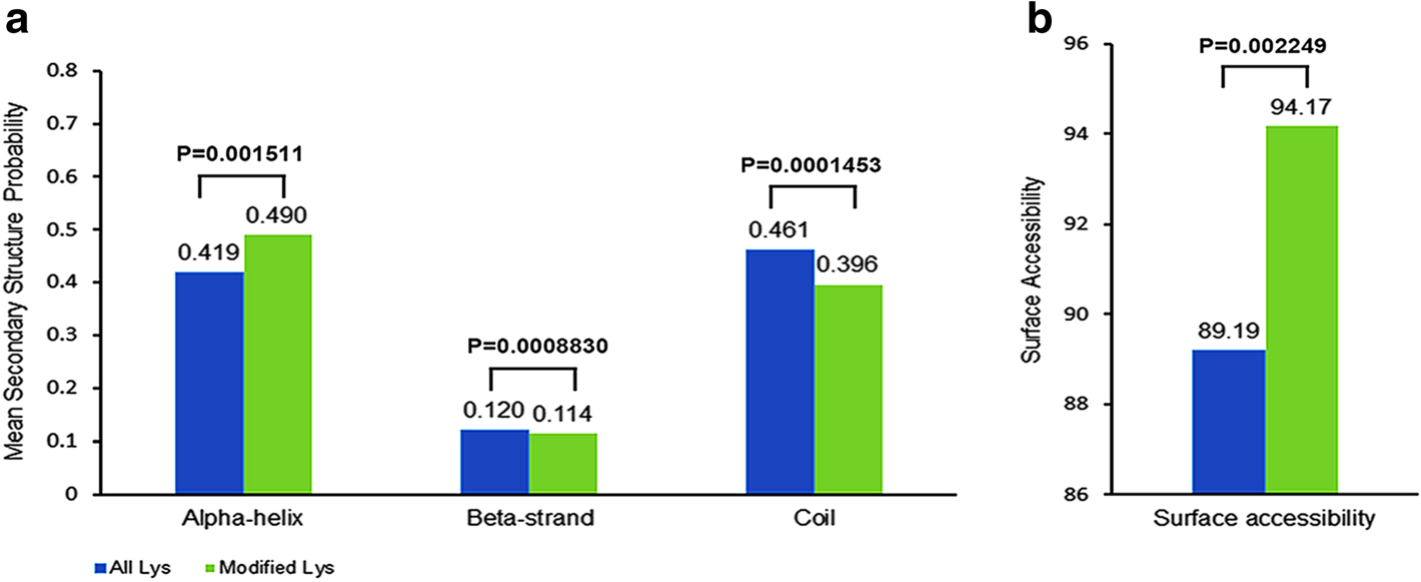
The distribution of the lysines in the protein secondary structures and their surface accessibility. (**a**) The protein secondary structures. (**b**) The surface accessibility. Succinylated lysines and all lysines were compared in the context of their protein secondary structures (α helix, β strand, and coil) and their relative surface accessibility (RSA). Significance was calculated by Wilcoxon rank-sum test. *P*< 0.05 was considered significant

We predicted the secondary structure features of the Ksucc sites by using NetSurfP. The secondary structures of succinylated lysines and all lysines were compared (Fig. 5). In our study, succinylated lysine residues were more frequently located on α-helix, and less frequently located on β-strand and coil, as compared with all lysine residues. The similar preferences were observed in rat that the succinylated lysine residues were moderately biased occurring on α-helix, and moderate biased against on β-strand and coil regions [33]. Furthermore, the succinylated sites tended to be exposed on the protein surface, as compared with all lysine residues.

### Difference in succinylation between the conidia and mycelia growth phases

As shown in the Venn diagram (Fig. 1b), 87% of the succinylated sites and 78% of the succinylated proteins were growth stage-specific. Although the succinylomes were markedly different in the conidia and mycelia phases, GO classification showed that the percentages of succinylated proteins in the majority of the categories were similar (Additional file 1: Figures S3-S5). This observation suggested the conserved roles of succinylation in different growth stages. However, some differences were found, as indicated below.

**Fig. 6:**
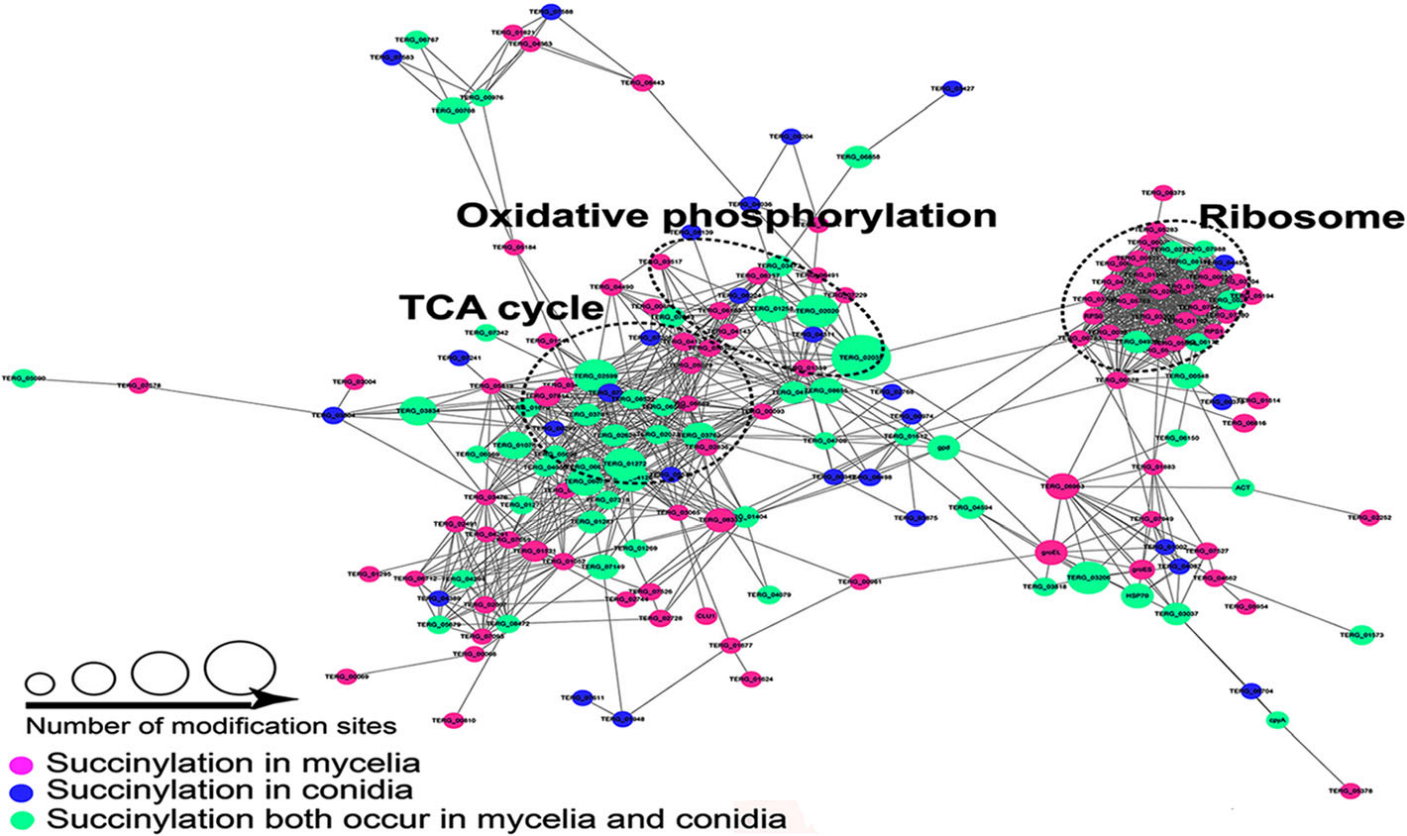
The PPI networks of the succinylated proteins. The top three enriched pathways are surrounded by a dotted circle. The *pink*, *blue*, and *green dots* indicate the succinylated proteins that were identified in only the mycelia stage, identified in only the conidia stage and both the mycelia and conidia stages, respectively

We analyzed the protein-protein interaction (PPI) networks of all the identified succinylated proteins by using the Cytoscape software (Fig. 6). Twelve clusters were significantly enriched (Additional file 10: Table S9). The top three clusters are shown in the Additional file 1: Figure S6. The most abundant cluster is involved in the ribosome complex, which has 29 protein nodes. Nevertheless, only one succinylated ribosomal protein (TERG_04450T0, 60S ribosomal protein L23) was specific to the conidia stage, whereas 6 succinylated ribosomal proteins were shared by the conidia and mycelia stages, and 22 (76%) succinylated ribosomal proteins were specific to the mycelia stage. We cannot conclusively determine whether this low level of succinylation of ribosomal proteins in the conidia stage was due to the reduced abundance of ribosomal proteins in dormant conidia, which are rarely identified by mass spectrometry analysis. In the proteomic analysis of dormant *T. rubrum* conidia, 56 proteins were identified as structural components of ribosomes, which accounted for 5% of all the identified proteins [12]. This result excluded the possibility that most ribosomal proteins were not identified in MS-based proteomic analyses of conidia. In addition, high levels of free ribosomes were also observed in the dormant conidia of *N. crassa* [34]. These free ribosomes immediately associate with a pre-existing pool of mRNA in the presence of a carbon source and initiate germination. On the basis of these observations, succinylation rarely modifies free ribosomes but heavily modifies polysomes during translation, thus suggesting that succinylation is essential for the process of translation.

**Fig. 7:**
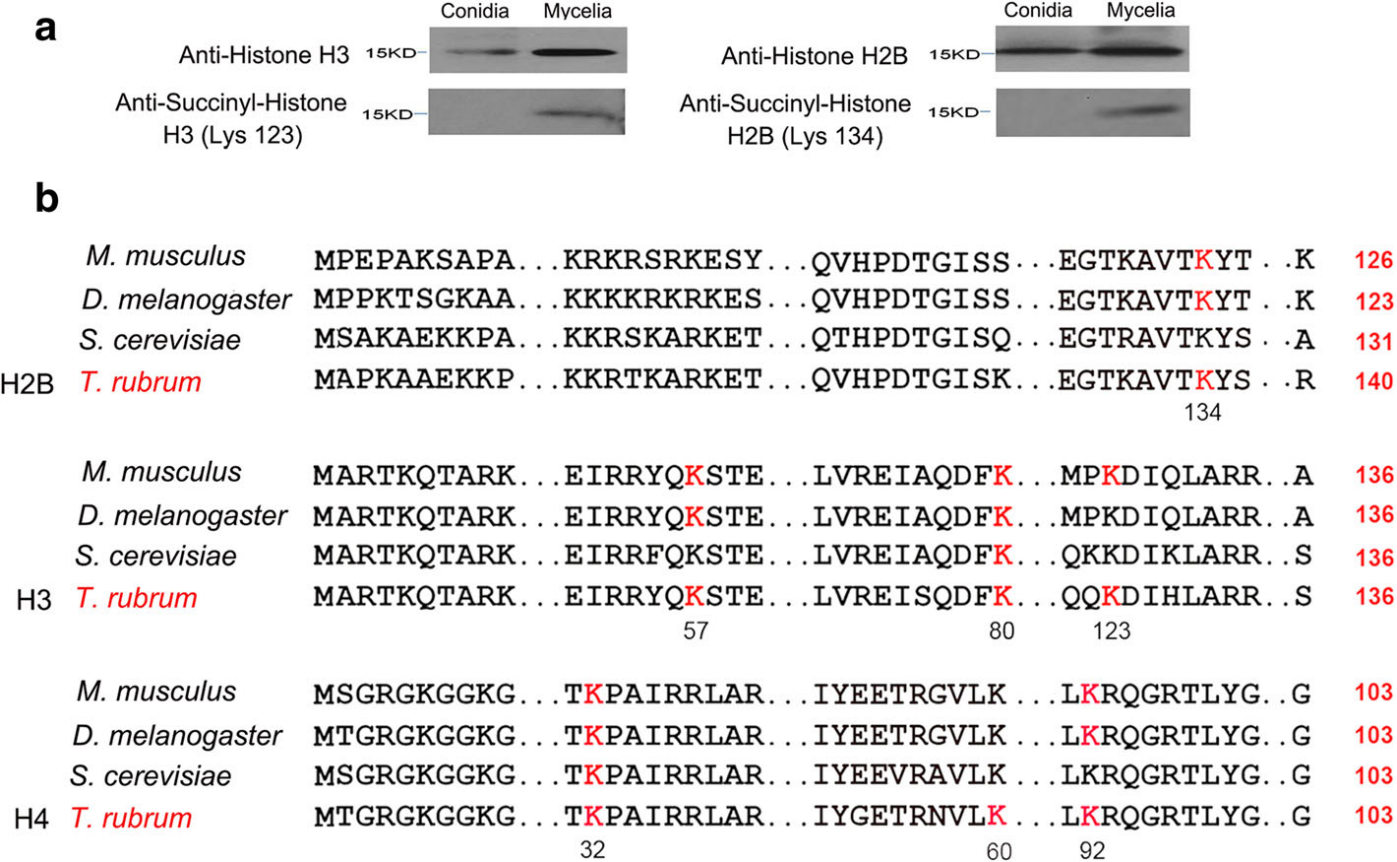
The succinylation modified sites on histones. **a** The western blotting analysis of two succinylated sites, H3K123 and H2BK134. **b** The identified succinylated sites on histones H2B, H3 and H4 in *T. rubrum* and the conservation of these sites in *M. musculus*, *D. melanogaster* and *S. cerevisiae*. The succinylated sites are shown in *bold red font*

Histone PTMs play a crucial role in epigenetic regulation. The contribution of histone acetylation to pathogenicity has been shown in both *C. albicans* [35] and *C. neoformans* [36]. Significant biological consequences of histone succinylation have also been shown. For example, H3K122 succinylation has been shown to be associated with chromatin accessibility [37]. We identified 7 succinylated sites on the core histones. Only one succinylated site (H3K80) was specific to the conidia stage, and 6 sites (H3K57, H3K123, H4K32, H4K60, H4K92 and H2BK134) were specific to the mycelia stage, thus suggesting that Ksucc participates differently in the epigenetic regulation between these two stages. We used two site-specific succinyl-histone antibodies to validate the results of mass spectrometry through western blotting analysis (Fig. 7a). The results showed that both of these sites (H3K123 and H2BK134) were identified in only mycelia. In addition, 6 sites (H3K57, H3K80, H3K123, H4K32, H4K92 and H2BK134) were conserved in other species [25] (Fig. 7b). Currently, H4K60 has not been reported to be succinylated at this site in the CPLM database [25]. This observation expands knowledge of Ksucc sites in histones.

We used the motif-X program to search for the sequence features surrounding the succinylated lysines. Two sequence patterns were defined as conserved succinylation site motifs (EK_succ_ and VK_succ_) in the conidia stage, and 4 conserved motifs (K_succ_Y, FK_succ_, LK_succ_ and K_succ_*L) were defined in the mycelia stage (Additional file 1: Figure S7). As shown in *V. parahaemolyticus* [38] and *T. gondii* [3], lysine (K) had the lowest frequency at the −1 and +2 positions. As shown in our heat map (Additional file 1: Figure S8), a similar preference was present for lysine in both stages. In addition, in *E. coli* [22] and *M. tuberculosis* [39], the region flanking the Ksucc site showed a strong preference for the acidic amino acids aspartic acid (D) and glutamic acid (E). In the conidia phase, the amino acid residues D and E were frequently present in the regions surrounding the Ksucc sites, but in the mycelia phase, this preference was not found. In addition, leucine (L) at the −1 and +2 positions, tyrosine (Y) at the +1 position, and glutamine (Q) at the −3, −1, and +1 positions were more abundant in the mycelia phase than in the conidia phase. These positional preferences for amino acid sequences surrounding the Ksucc sites reflected the preferred substrate sequences for lysine succinyltransferase, which catalyze succinylation. The different sequence features in the vicinity of Ksucc sites between these two different stages prompt the question of whether succinylation is catalyzed by enzymes that are specific to each growth phase.

## Conclusions

In this study, we presented the first in-depth analysis of the succinylome of *T. rubrum*, which is considered a good model for studying anthropic pathogenic filamentous fungi. We used a combination of highly accurate nano-LC-MS/MS and an affinity enrichment approach to identify the succinylated peptides. We identified 569 succinylated sites in 284 proteins in *T. rubrum*. The succinylome of *T. rubrum* targeted a broad range of proteins but was most significantly enriched in proteins involved in metabolic pathways. According to studies of lysine acylation, such as methylation and acetylation, all of these acylations have important roles in regulating metabolic enzymes [40, 41]. Thus, succinylation may work together with other types of PTMs in affecting structure and functions of protein involved in metabolism. In addition, succinylation, as compared with methylation and acetylation, alters the charge status more (inducing a two-charge shift in the substrate residues) and has a larger structural moiety. We wondered whether the greater changes imposed by succinylation might lead to a more significant alteration in protein structure and function. The differences in the specific roles of succinylation and other types of acylation are still unclear, and the future explorations of these differences will be intriguing. In addition, 24 pathogenicity-related proteins reported in *T. rubrum* and other pathogenic fungi were found to be succinylated. Thus, it will be interesting to investigate whether a relationship exists between succinylation and pathogenicity.

*T. rubrum* is a filamentous fungus with two major growth stages. The conidia stage is in dormancy and has a relative low level of metabolism, but it is a competent state that harbors a set of metabolic enzymes to degrade storage compounds [42]. In this stage, the enzymes necessary for germination and a pool of pre-existing mRNAs and ribosome are present, thus enabling rapid initiation of germination and mycelial growth in the presence of carbon sources [42, 43]. In the mycelia stage, both metabolism and protein synthesis are in an active state; thus, respiratory metabolism-related enzymes, mitochondrial proteins and translation-relating proteins are all up-regulated [11, 43]. Comparison of the succinylome of the conidia and mycelia stages revealed that most succinylated proteins and sites were different between the two growth stages. GO classification revealed that metabolism was the largest functional group represented by the succinylated proteins. The number of succinylated proteins involved in metabolism increased from 91 in the conidia stage to 131 in the mycelia stage, but the percentage of metabolism-relating proteins in the conidia and mycelia stages were almost the same (Additional file 1: Figure S3). Of these succinylated proteins involved in metabolism, 45 proteins were specific to conidia stage, and 85 proteins were specific to mycelia stage. In spite of this, our study showed that succinylated proteins participate in similar metabolism pathways between the two growth stages, such as glycolysis, the TCA cycle, and oxidative phosphorylation (Additional file 11: Table S10). However, some differences were found. Succinylation was much more abundant in ribosomal proteins during the vigorous growth period of the mycelia stage than in the dormant conidia stage (Fig. 6), suggesting that succinylation heavily modifies the ribosome only when translation is occurring. Additionally, the succinylated sites on core histones were different between the conidia and mycelia stages, and the succinylated site H4K60 has not been reported in the CPLM database to date.

Succinylation is regulated by two types of enzymes with opposing activities, lysine succinyltransferase and desuccinylases. So far no enzyme that catalyze succinylation and desuccinylation have been reported in fungi. Histone acetyltransferases p300 is the only enzyme displayed lysine succinyltransferase activity identified to date [44]. Because of the structural similarity of these short-chain acyl-CoAs (like malonyl-CoA, succinyl-CoA, and glutaryl-CoA) to acetyl-CoA, it has been suggested that the promiscuous acyltransferases activity catalyzed by acetyltransferases [44]. In *T. rubrum*, 18 proteins have been annotated as acetyltransferase (TERG_00136T0, TERG_00160T0, TERG_00920T0, TERG_00960T0, TERG_02546T0, TERG_03442T0, TERG_03711T0, TERG_04055T0, TERG_04687T0, TERG_04983T0, TERG_05174T0, TERG_05450T0, TERG_05561T0, TERG_06411T0, TERG_06572T0, TERG_07217T0, TERG_07375T0 and TERG_07548T0). Further experiments are needed to test whether one or several of these acetyltransferases possess succinyltransferase activities, or whether another succinyltransferase exists in *T. rubrum*.

Sirtuins, also known as Sir2 (Silent information regulator 2) proteins, are a class of NAD^+^-dependent deacetylases. In mammalian cells, seven sirtuins (SIRT1–7) have been identified. It has been shown that SIRT3–5 and SIRT7 exhibit desuccinylases activity [44]. In yeast, five Sir2 proteins have been identified (Sir2p and Hst1–4) [45]. Except for acetylation, some Sir2 proteins can catalyze other types of lysine acylation [46]. For example, in yeast, Hst2 has a greater affinity for binding propionyl-lysine and butyryl-lysine than acetyl-lysine [47]. In *T. rubrum*, 5 potential deacylases have been annotated as Sir2 family histone deacetylases (TERG_03010T0, TERG_03268T0, TERG_05234T0, TERG_06970T0 and TERG_07330T0). Further experiments will be needed to investigate the activities of these Sir2 proteins. We wondered whether one of these deacetylases might play a role in desuccinylase activities or whether another desuccinylase exists in these dermatophytes. In addition, our study showed the different amino acid sequence features surrounding the succinylated sites between the two growth stages, suggesting that the enzymes that regulate succinylation may function in a growth-phase dependent manner.

Our dataset provides a valuable resource for future studies on the function and mechanism of succinylation, as well as its roles in physiological and pathogenic processes in *T. rubrum*. The findings described here should aid in understanding the effects of succinylation in dermatophytes with homology to *T. rubrum*, and consequently understanding of these clinically important pathogenic filamentous fungi. Moreover, these data should support the development of improved therapeutics for treating dermatophyte infections in the near future.

## Methods

### Strain and culture

The fungus *T. rubrum* (strain BMU 01672) was obtained from the Research Center for Medical Mycology at Peking University in Beijing, China. The conidia phase of *T. rubrum* was obtained by culturing the fungus on potato glucose agar at 28 °C for 3 weeks. The mycelia phase of *T. rubrum* was obtained by culturing the fungus in Sabouraud Liquid Medium (containing 20 g of glucose and 10 g of peptone in 1 L of distilled water) at 28 °C with constant shaking (200 rpm).

### Protein extraction

The fungal cells were ground in liquid nitrogen and then suspended in lysis buffer (8 M urea, 10 mM dithiothreitol (DTT), 50 mM nicotinamide (NAM), 3 μM trichostatin A (TSA) and 0.1% protease inhibitor cocktail). The supernatant was collected by centrifugation. Then, the proteins were precipitated with 15% trichloroacetic acid (TCA) at −20 °C for 2 h. The protein precipitate was then washed with cold acetone three times. After the evaporation of the residual acetone, the precipitated protein was re-dissolved in 8 M urea and 100 mM NH_4_HCO_3_ (pH 8.0). The protein concentration was determined with a 2-D Quant Kit (GE Healthcare) according to the manufacturer’s instructions.

### Trypsin digestion

The proteins were reduced by incubation with 10 mM DTT for 1 h at 37 °C and alkylated with 20 mM iodoacetamide (IAA) for 45 min at room temperature (RT) in the dark. The proteins were digested with trypsin (Promega) at a trypsin/protein ratio of 1:50 (*w*/w) overnight at 37 °C.

### HPLC fractionation

The digested peptides were fractionated into 80 fractions by reverse-phase HPLC using an Agilent 300Extend-C18 column (5-μm particles, 4.6-mm ID, 250-mm long) with a gradient of 2% to 60% acetonitrile in 10 mM ammonium bicarbonate (pH 10). Then, the fractionated peptides were combined into 3 fractions and dried with vacuum centrifugation.

### Affinity enrichment of the succinylated peptides

The peptides were dissolved in NETN buffer (100 mM NaCl, 1 mM EDTA, 50 mM Tris-HCl, and 0.5% NP-40, pH 8.0) and then incubated with pre-conjugated pan-antisuccinyl lysine agarose beads (PTM Biolabs) overnight at 4 °C with gentle shaking. After incubation, the beads were washed carefully with NETN buffer four times and with ddH_2_O twice. The peptides bound to the beads were eluted with 0.1% trifluoroacetic acid (TFA) and dried with vacuum centrifugation. The peptides were re-dissolved in 0.1% formic acid (FA) and desalted with C18 ZipTips (Millipore).

### LC-MS/MS analysis

The peptides were separated using an EASY-nLC 1000 UPLC system equipped with a reverse-phase pre-column (Acclaim PepMap 100, Thermo Scientific) and a reverse-phase analytical column (Acclaim PepMap RSLC, Thermo Scientific). The flow rate was 350 nL/min, and the gradient was 5% to 25% solvent B (0.1% FA in 98% ACN) for 26 min, 25% to 40% for 8 min, 40% to 80% in 3 min and finally holding at 80% for 3 min.

The peptides that eluted from the column were subjected to an NSI source followed by tandem mass spectrometry (MS/MS) using a Q Exactive™ Plus (Thermo Fisher Scientific) instrument. The mass window was 350 to 1800 m/z for MS scans. A full range mass scan was acquired with a high resolution of 70,000 and was followed by 20 data-dependent MS/MS scans at a resolution of 17,500. The top 20 precursor ions above a threshold ion count of 5E^3^ with a 15-s dynamic exclusion were selected for MS/MS using higher energy C-trap dissociation (HCD) at 28% normalized collision energy. Three biological replicates were performed.

### Database search

The raw MS/MS data were compared with the *T. rubrum* protein database version 2 (https://archive.broadinstitute.org/ftp/pub/annotation/fungi/dermatophytes/genomes/trichophyton_rubrum_cbs_118892/) (containing 11,418 sequences, commonly observed contaminants were appended to the database) and concatenated with a reverse decoy database using MaxQuant with the integrated Andromeda search engine (v.1.4.1.2).

The database searches were performed with the following parameters: cleavage enzyme: trypsin; maximum missed cleavages: 4; maximum modifications per peptide: 5; maximum charge per peptide: 5; mass tolerance for precursor ions: 5 ppm; mass tolerance for fragment ions: 0.02 Da; static modifications: carbamidomethylation of cysteine (+57.0215 Da); dynamic modifications: oxidation of Met (+15.995 Da), succinylation of lysine and succinylation on the protein N-terminus (+100.0160 Da); and minimum peptide length: 7. The false discovery rate (FDR) cut-off of <1% was specified for the protein, peptide and modification sites. The site localization probability was set to >0.75. All the other parameters in MaxQuant were set to default values. Peptide scores above 40 were considered for further analysis. Only leading proteins were reported for each MS/MS search.

### Western blotting

Cell lysates from the conidia and mycelia stages were loaded on a 12% SDS-PAGE gel. The proteins were transferred to PVDF membranes (Bio-Rad Laboratories) and incubated in blocking buffer for 2 h. Then, the membranes were incubated with pan anti-succinyl antibodies, anti-histone antibodies (anti-H2B and anti-H3) and histone Ksucc site-specific antibodies (anti-H2BK134succ and anti-H3K123succ) (PTM Biolabs) at 4 °C overnight and subsequently incubated with (HRP)-conjugated goat anti-rabbit antibody (Pierce) for 2 h at RT.

### Bioinformatics analysis

The identified succinylated proteins were compared against the GO database by using Blast2GO. The subcellular localization was predicted using WoLF PSORT software. For the pathway analysis, the proteins were annotated using the KEGG online service tool KAAS (KEGG Automatic Annotation Server), and the results were mapped on the KEGG pathway database by using the KEGG online service tool KEGG Mapper.

Two-tailed Fisher’s exact tests were applied to each category to obtain *p* values for the analysis of the functional enrichment or depletion of the identified proteins compared with all database proteins. Terms with *p* values less than 0.01 were considered significant.

The protein − protein interaction (PPI) network was obtained from the STRING database (version 9.1); the confidence score for all the obtained interactions was at least 0.7 (high confidence). The PPI network was visualized using Cytoscape (version 2.8.3), and densely connected regions in this network were further analyzed by using a graph created with the clustering algorithm “Molecular Complex Detection” (MCODE).

Amino acid sequence models (10 amino acids upstream and downstream of the succinylated lysine) were analyzed by using motif-X to analyze the motifs containing the succinylated lysines. The *T. rubrum* protein sequence database was used as the background database parameter, and other parameters used the default settings.

The secondary structure properties (alpha-helix, beta-strand and coil) and surface accessibility of the lysine residues were determined using NetSurfP. Significance was calculated by Wilcoxon rank-sum test. *P* < 0.05 was considered significant.

## Additional files

Additional file 1: Figures S1-S8. All supplementary figures. (PDF 8848 kb)

Additional file 2: Table S1. The list of all the identified succinylated peptides and the corresponding proteins in each replication of conidia and mycelia stages. (XLSX 140 kb)

Additional file 3: Table S2. GO classification of the succinylated proteins. (XLSX 12 kb)

Additional file 4: Table S3. Subcellular localization of the succinylated proteins. (XLSX 11 kb)

Additional file 5: Table S4. The proportion of succinylated sites per protein for mitochondrial and non-mitochondrial proteins. (XLSX 9 kb)

Additional file 6: Table S5. GO enrichment analysis of the succinylated proteins. (XLSX 13 kb)

Additional file 7: Table S6. KEGG enrichment analysis of the succinylated proteins. (XLSX 14 kb)

Additional file 8: Table S7. The succinylated enzymes and modified sites in TCA cycle. (XLSX 11 kb)

Additional file 9: Table S8. The succinylated enzymes and modified sites in oxidative phosphorylation. (XLSX 11 kb)

Additional file 10: Table S9. The PPI network analysis of the succinylated proteins. (XLSX 185 kb)

Additional file 11: Table S10. The succinylated proteins involved in metabolism in the conidia and mycelia stages of *T. rubrum*. (XLSX 44 kb)

## Abbreviations

FDR: False discovery rate
GO: Gene ontology
KEGG: Kyoto Encyclopedia of Genes and Genomes
Ksucc: Lysine succinylation
LC-MS/MS: Liquid chromatography-tandem mass spectrometry
PPIs: Protein–protein Interactions
PTM: Post-translational modification
*T. rubrum*: *Trichophyton rubrum*
