# Supplementary material for: The first succinylome profile of Trichophyton rubrum reveals lysine succinylation on proteins involved in various key cellular processes
Source: BMC Genomics. 2017 Aug 4;18:577. doi: 10.1186/s12864-017-3977-y (PMC5545033; doi:10.1186/s12864-017-3977-y)
Supplement: Supplementary file 1 — All supplementary figures. (PDF 8848 kb) [file 12864_2017_3977_MOESM1_ESM.pdf]

Supplementary Figures and Legends

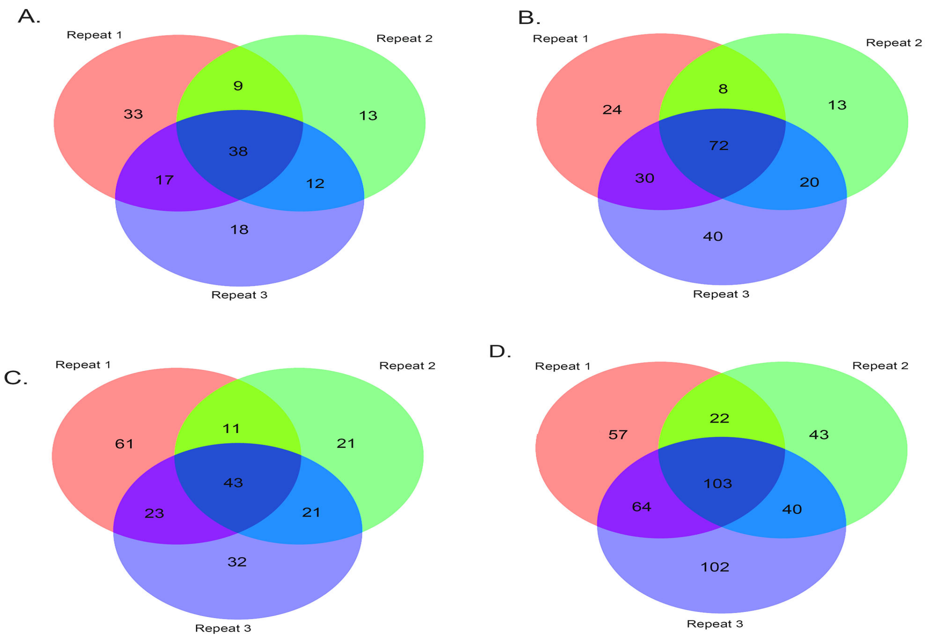

Figure S1. Venn diagram of the succinylated proteins and sites identified in each replication. (A) Succinylated proteins identified in conidia stage. (B) Succinylated proteins identified in mycelia stage. (C)Succinylated sites identified in conidia stage. (D) Succinylated sites identified in mycelia stage.

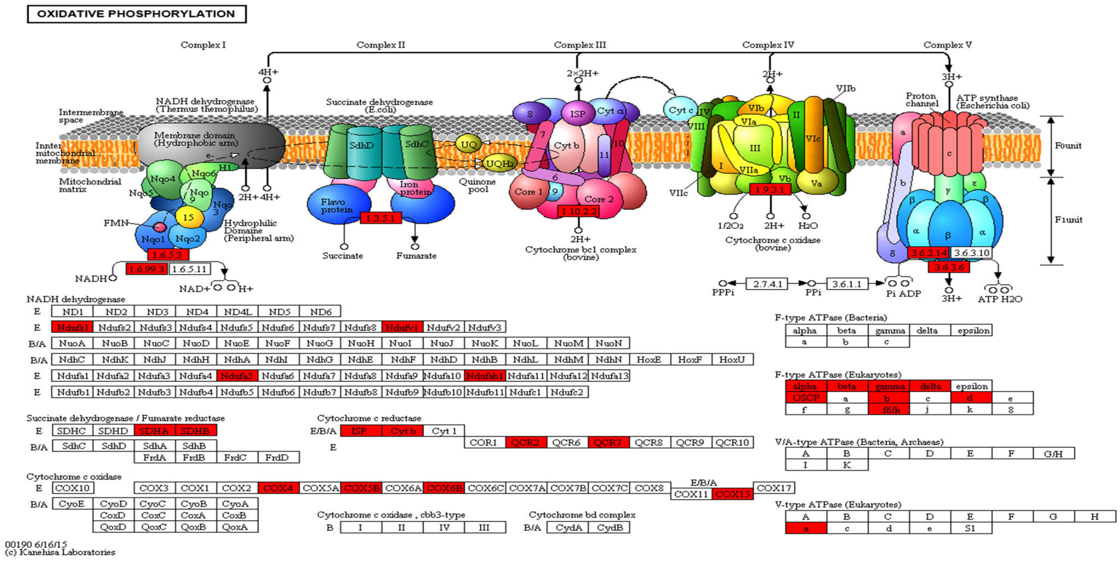

Figure S2. Succinylated proteins involved in the oxidative phosphorylation. The succinylated proteins are highlighted in red.

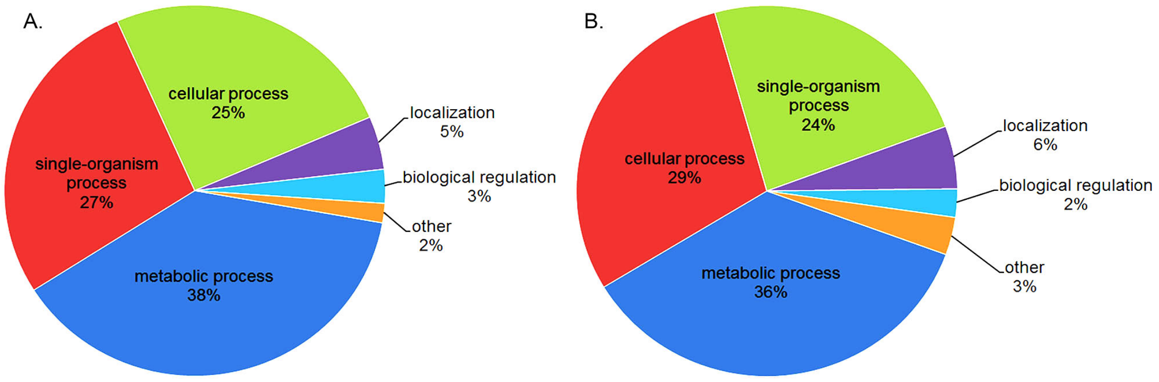

Figure S3. GO classification of the succinylated proteins based on biological process in the conidia and mycelia stages. (A) Conidia stage. (B) Mycelia stage.

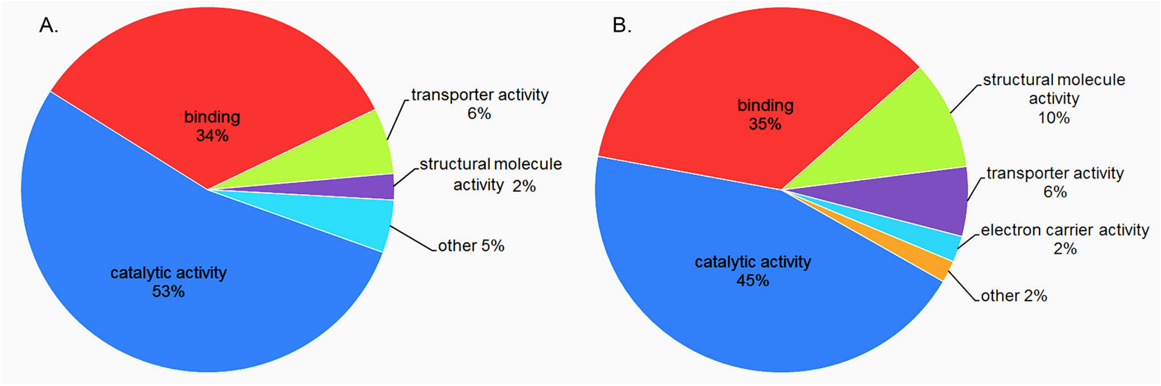

Figure S4. GO classification of the succinylated proteins based on molecular function in the conidia and mycelia stages. (A) Conidia stage. (B) Mycelia stage.

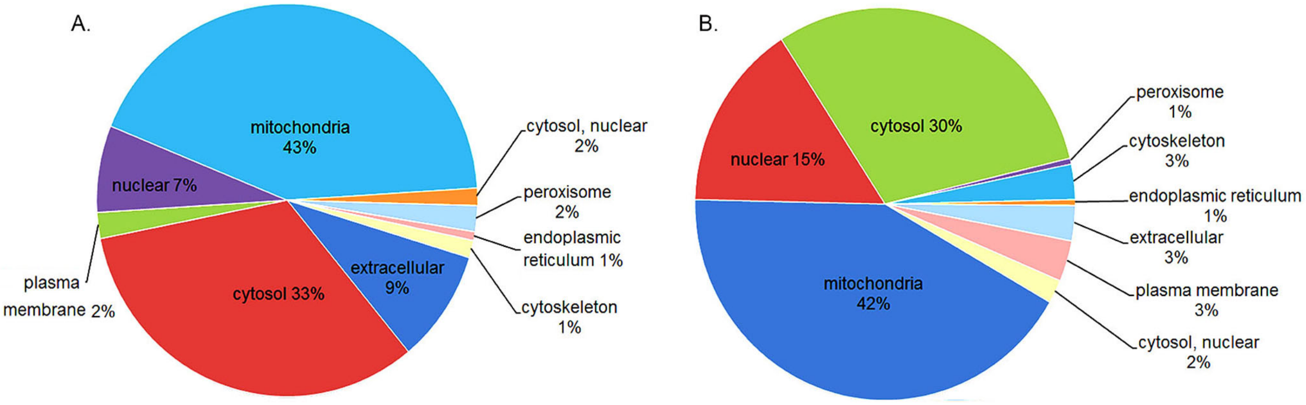

Figure S5. Classification of the succinylated proteins based on subcellular location analysis in the conidia and mycelia stages. (A) Conidia stage. (B) Mycelia stage.

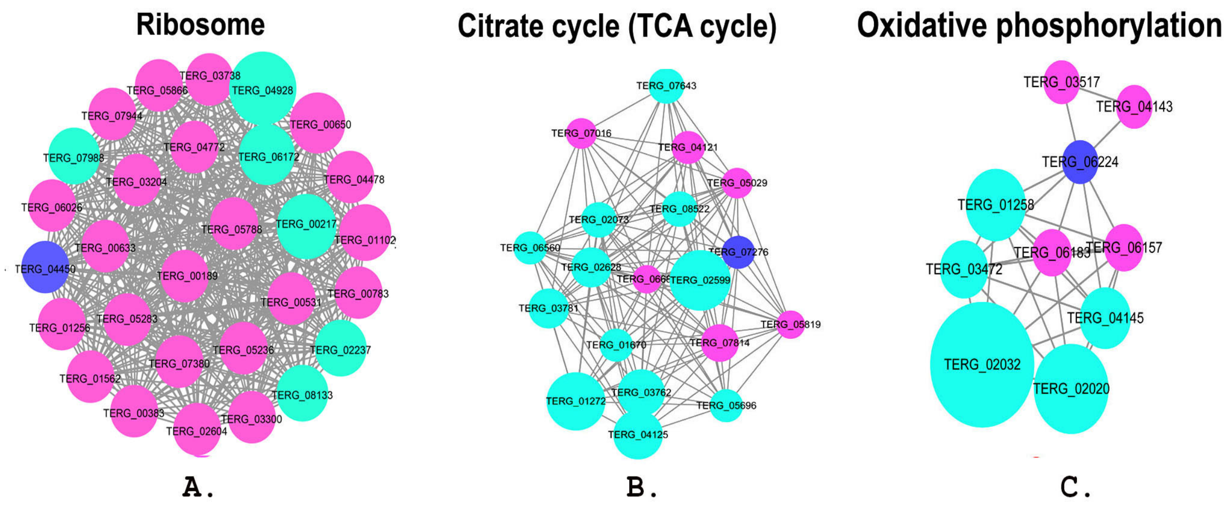

Figure S6. The top three clusters in the PPI network of the succinylated proteins. (A) Ribosome. (B) TCA cycle. (C) Oxidative phosphorylation. The pink, blue, and green dots indicate the succinylated proteins that were only identified in the mycelia stage, only identified in the conidia stage and shared by the mycelia and conidia stages, respectively.

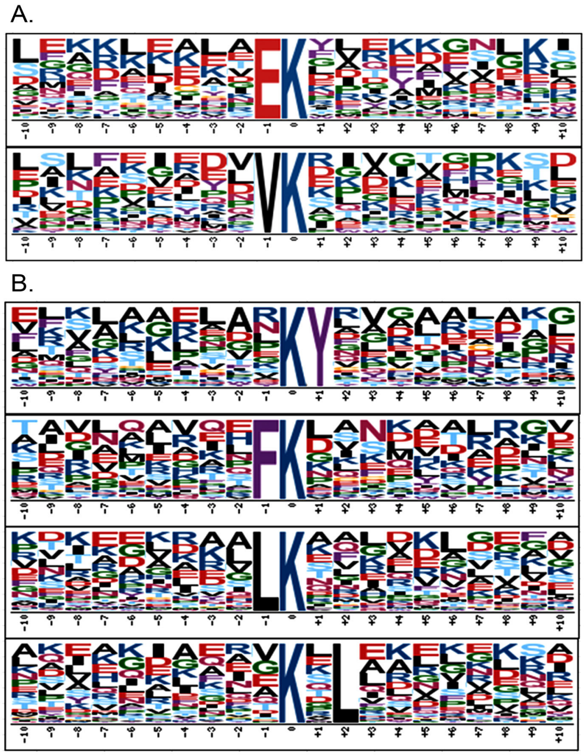

Figure S7. The sequence motifs of the succinylated sites in the conidia and mycelia stages. (A) Conidia stage. (B) Mycelia stage. Motifs with a significant difference of $P < 0.0001$ are shown.

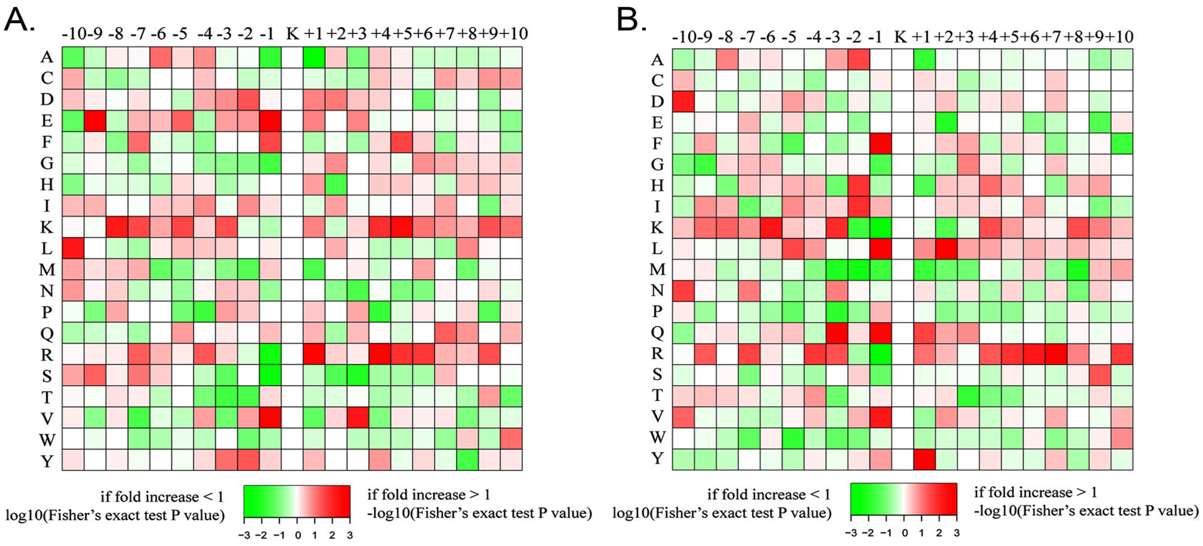

Figure S8. Heat maps showing the frequency of use of the amino acids flanking the succinylation sites in the conidia and mycelia stages. (A) Conidia stage. (B) Mycelia stage. The colors represent the enrichment (red) or depletion (green) of amino acids in specific positions flanking the succinylated sites.
